# Detection and Quantification of Adulterated Beef and Mutton Products by Multiplex Droplet Digital PCR

**DOI:** 10.3390/foods11193034

**Published:** 2022-09-30

**Authors:** Chuan He, Lan Bai, Yifan Chen, Wei Jiang, Junwei Jia, Aihu Pan, Beibei Lv, Xiao Wu

**Affiliations:** 1 Biotechnology Research Institute, Shanghai Academy of Agricultural Sciences, Shanghai 201106, China; 2 Shanghai Key Laboratory of Agricultural Genetics and Breeding, Shanghai Academy of Agricultural Sciences, Shanghai 201106, China; 3 Crops Ecological Environment Security Inspection and Supervision Center (Shanghai), Ministry of Agriculture and Rural Affairs, Shanghai 201106, China

**Keywords:** meat adulteration, food quality control, quantification, nucleic acid detection, droplet digital PCR

## Abstract

In order to seek high profit, businesses mix beef and mutton with cheap meat, such as duck, pork, and chicken. Five pairs of primers were designed for quintuple droplet digital PCR (qddPCR) of specific genomic regions from five selected species and specificity and amplification efficiency were determined. The mixed DNA template with an equal copy number was used for detecting the accuracy and limit of multiplex PCR. The results showed that the primers and probes of the five selected species had good specificity with the minimum number of detection copies: 0.15 copies/µL beef (*Bos taurus*), 0.28 copies/μL duck (*Anas platyrhynchos*), 0.37 copies/μL pork (*Sus scrofa*), 0.39 copies/μL chicken (*Gallus gallus*), and 0.41 copies/μL mutton (*Ovis aries*), respectively. The five sets of primers and probes could quickly judge whether the specified meat components existed in the food commodities.

## 1. Introduction

Beef, mutton, pork, chicken, and duck are major meat products consumed in China. Compared with other meats, beef and mutton are more expensive, making unscrupulous sellers mix cheaper meat, such as pork, chicken, and duck into beef and mutton products, and even declare pork, chicken, and duck to be beef and mutton to earn more profits [1]. It was reported that certain countries also had similar food-safety incidents passing off horse meat as beef [2]. As a result, international food enterprises started to strengthen the detection of ingredients. Even though national food-safety standards in many countries require meat products to be labeled with accurate and detailed information, including the composition and percentage of meat from different species [3,4,5], it is difficult for consumers to identify red meat from the appearance of processed meat, and these problems seriously interfere with the safety of meat products [6,7,8]. Exported meat products also need strict component testing to ensure quality. Detecting animal species in meat products is important, as substitution can be a problem for people with food allergies as well as for those who do not eat certain species on religious grounds. To ensure food safety and fair trade in both local and international markets, efficient and accurate methods are needed to detect and quantify adulterated meat products.

Real-time quantitative polymerase chain reaction (qPCR) is a common technique for identifying the composition of meat products. The advantages of qPCR are high sensitivity and speed, but it is difficult to determine whether adulterated ingredients are intentionally added or unintentionally contaminated. This is because if the adulteration amount is determined by qPCR, a standard curve must be constructed to prepare the standard sample and then calculate the adulteration percentage. Therefore, it is urgently needed to establish a precise, rapid, and simple method to quantify other animal-derived adulterated ingredients in beef and mutton products.

Droplet digital PCR (ddPCR) is a novel method for precise quantification of nucleic acids that has been applied in the identification and quantification of plant and animal species in recent years [9,10]. The PCR mixture is partitioned into thousands of nanoliter-sized droplet reactions by the droplet generator before the traditional PCR amplification [7,11,12]. Each droplet is an independent PCR and is identified as a positive or negative signal based on its fluorescence signal. Subsequently, these fluorescent signals are counted and recorded by the droplet reader. The interpretation of droplets with fluorescence signal is 1 and that of droplets without fluorescence signal 0. According to the Poisson distribution principle, the initial copy number or concentration of the target molecule can be derived from the number and proportion of positive droplets.

The significant advantage of ddPCR over qPCR is that the concentration of the sample can be determined directly without a standard curve. Therefore, ddPCR is suitable for the quantitative detection of animal-derived components [13].

Currently, ddPCR is applied to detect animal-derived ingredients. For example, ddPCR was used to quantify pork materials in beef products [9,14], assay pork incorporated into mutton products [4], and determine meat proportions of sausages containing meat from chicken, turkey, horse, cow, pig, and sheep [15]. However, the detection of multiple animal-derived ingredients in a single sample remains immature. Therefore, a multiple ddPCR detection method was established for beef and mutton products in order to provide technical support for the rapid and quantitative detection of animal-derived ingredients.

## 2. Materials and Methods

### 2.1. Meat-Sample Preparation

Fresh beef, mutton, pork, chicken and duck were purchased at the Longshang agricultural trade market. All samples were separately minced in a blender. Then, powdered samples were frozen at −20 °C [9].

### 2.2. DNA Extraction

DNA was extracted from 100 mg powdered samples using a genomic DNA-extraction kit (Shanghai Biotechnology Co., Ltd., Shanghai, China). The quality of extracted DNA was analyzed by agarose gel electrophoresis and the DNA concentration of each sample was measured with a NanoDrop 2000 spectrophotometer (Thermo Scientific, Waltham, MA, USA).

### 2.3. Primers and Probes

For the detection and quantification of beef (*Bos taurus*), mutton (*Ovis aries*) and duck (*Anas platyrhynchos)*, the *Bos taurus* beta-actin (ACTB) gene (GenBank accession number: EH170825), *Ovis aries* gene (GenBank accession number: MW364895.1), and *Anas platyrhynchos* gene (GenBank accession number: MF069251) were selected as the target sequences. The primers and probes of *Bos taurus*, *Ovis aries* and *Anas platyrhynchos* were designed by Oligo6.0, and then validated for specificity and homology by BLAST against the entire GenBank database [16]. According to media reports [1], common foods such as beef and mutton are always counterfeited with other type of meats. Therefore, beef and mutton were considered originals and pork, chicken, and duck considered adulterants in the research. Probes for beef and mutton were labeled with HEX fluorophore and minor groove binder (MGB) and probes for pork, duck and chicken were labeled with FAM fluorophore and MGB. The primers and probes for pork (*Sus scrofa*) and chicken (*Gallus gallus*) were designed according to published sequences [17] and were synthesized by Shanghai Biotechnology Co., Ltd. (Table 1).

### 2.4. Specificity Assays

The primers and probes were applied in qPCR to assess the specificity and amplification efficiency using DNA samples from beef, mutton, pork, chicken and duck. Standard curves were constructed by diluting the DNA samples to ×1, ×10, ×100, ×1000, ×10,000, ×100,000 and ×1,000,000 gradients.

Each 20 μL reaction mixture of qPCR was prepared as follows: 10 μL 2 × TaqMan universal mix (BBI), 6.8 μL ddH_2_O, 1 μL DNA template, and primers and probes at a concentration of 200 nmol/L. The reaction conditions were 94 °C for 3 min, 95 °C for 5 s, 60 °C for 15 s, and 72 °C for 30 s, totaling 45 cycles. Three parallel reactions were performed for each sample.

### 2.5. Sensitivity Analysis of Primers and Probes

The DNA of meats (beef, mutton, pork, chicken and duck) was diluted to 20 ng/μL, 10 ng/μL, 5 ng/μL, 2 ng/μL, 1 ng/μL, 0.5 ng/μL, 0.1 ng/μL, and 0.01 ng/μL in turn, and the sensitivity and limit of detection (LOD) were assayed using five pairs of primers.

The 20 μL ddPCR mixture consisted of 1.8 μL forward and reverse primers (final concentration, 900 nM), 0.5 μL of the probe (final concentration, 250 nM), 10 μL of ddPCR Master Mix (Bio-Rad, Hercules, CA, USA), 1 μL of template DNA, and 4.9 μL of nuclease- and protease-free water (Thermo Scientific).

After the PCR, the droplet reading and analysis system can automatically read fluorescence signals in the droplet number, then calculate the copy number in the PCR system according to the Poisson distribution principle and divide the copy number by the volume of the PCR system to obtain the lowest copy number that can be detected by the ddPCR.

### 2.6. Fluorescence Interference

To assess fluorescence interference between the probes, a ddPCR system was evaluated using DNA derived from single-species samples or combined-species samples (beef, mutton, pork, chicken and duck). The deviation of the measured value relative to the true value was calculated from three measurements. Three independent experiments were performed.

### 2.7. Droplet Digital PCR Assay

The reaction mixture was divided into approximately 20,000 droplets using a QX200 droplet generator (Bio-Rad). PCR was performed in a C1000 thermal cycler (Bio-Rad) under the following conditions: 95 °C for 10 min, followed by 40 cycles of denaturation at 94 °C for 30 s and annealing/extension at 60 °C for 1 min, 98 °C for 10 min. The samples were then loaded into a QX200 droplet reader (Bio-Rad). Droplets were arranged in a single file to pass through a two-color detection system droplet by droplet. The droplet reader then counted which ones were positive and which negative and how many of each there were in the individual samples. The positive droplets exhibited increased fluorescence compared to the negative droplets. The QuantaSoft software measured the numbers of droplets that are positive and negative for each fluorophore in the sample. The fraction of positive droplets was then fitted to a Poisson distribution to determine the absolute initial copy number of the target DNA in the input reaction mixture in units of copies/µL.

Template mixing and selective addition of primers was adopted to detect the accuracy of single, double, triple, quadruple and quintuple ddPCR (Table 2). Samples of beef, mutton, pork, chicken and duck were diluted to a similar number of replicates. The DNA samples from beef and mutton were mixed in a 1:1 ratio. Meanwhile the DNA samples of pork, chicken and duck were mixed according to the copy number ratios of 1:1:1, 1:2:3 and 1:3:6, respectively.

### 2.8. Analysis of Samples of Known Composition

DNA samples from the food products containing more than two types of animal meat can be used for identification and quantification. However, some factors may affect DNA extraction and detection, including meat species, production processes, DNA degradation, tissue composition, and amplification efficiency. To assess the accuracy and applicability of ddPCR, ten meat samples of known composition were prepared and analyzed.

## 3. Results

### 3.1. Species Specificity

Amplification of DNA from different pure meat products (beef, mutton, pork, chicken and duck) was accomplished by qPCR technique in combination with appropriate specific primers and probes. Specific primers and probes amplified with the corresponding meat DNA template, forming an amplification curve without interference from other templates. The results showed that the selected primers and probes had positive species specificity.

### 3.2. Amplification Efficiency of Primers

By measuring the concentration and purity of the extracted DNA, the OD260/OD280 values were 1.83~1.87, all within the normal range of 1.8~2.0 (Table 3). Gradient dilution of five DNA samples were used as templates to construct a standard curve. The results showed that each pair of primers had high amplification efficiency (Table 4).

### 3.3. Sensitivity of Primers and Probes

**Figure 1:**
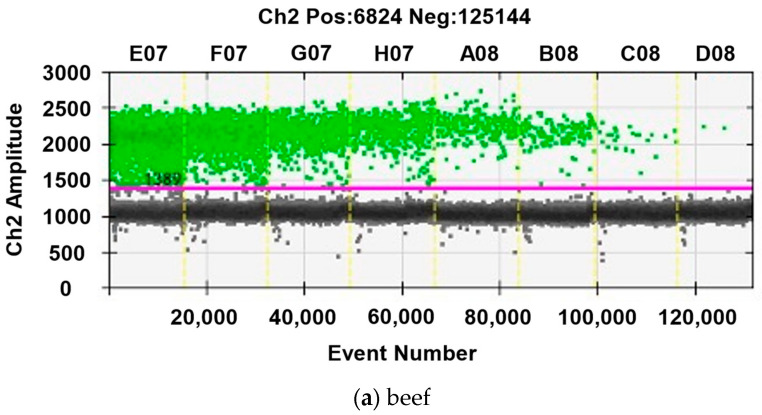

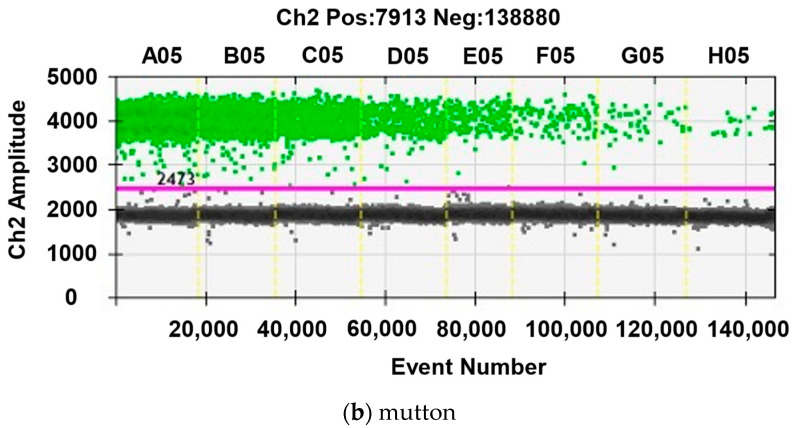
Scatterplot under different template concentrations, (**a**) beef, (**b**) mutton. Each droplet is plotted on the graph of fluorescence intensity versus droplet number. The eight columns represent serial dilution of each meat DNA. The amplitude graph shows positive (in green) and negative (in black) droplet populations. The threshold is set in between, as indicated by the purple line.

Visualization of the ddPCR system was performed for the determination of beef and mutton in channel 2 (Hex) (Figure 1). The number of copies of DNA was calculated by online software (http://cels.uri.edu/gsc/cndna.html (accessed on 24 September 2021)). The beef (*Bos taurus*) primers had the highest sensitivity with the lowest detection concentration of 0.15 copies/μL. As for the other primers, concentrations from low to high were 0.28 copies/μL duck (*Anas platyrhynchos*), 0.37 copies/μL pork (*Sus scrofa*), 0.39 copies/μL chicken (*Gallus gallus*), and 0.41 copies/μL mutton (*Ovis aries*).

### 3.4. Accuracy of Multiplex PCR

DNA templates from beef, mutton, pork, chicken and duck were diluted to concentrations with similar number of copies (342 copies/μL, 338 copies/μL, 376 copies/μL, 356 copies/μL, and 326 copies/μL, respectively) and were mixed in the same proportion.

The DNA samples and probes were added according to Table 2. To investigate the accuracy of multiplex PCR, the detection results of different primers and probes were compared based on DNA from beef and mutton as templates. The actual detection value was in good agreement with the expected value, and the detection values of adding *Bos taurus* and *Ovis aries* primers was equal to the sum of the detection values of adding *Bos taurus* or *Ovis aries* primers alone (Table 5). Similarly, the same abovementioned situation was found adding primers for pigs, ducks and chickens (Table 6).

### 3.5. Analysis of Samples of Known Composition

The DNA samples of beef and mutton (1:1) and pork, chicken, duck (1:1:1) were mixed again according to the proportion (Table 7). Theory value (copies/μL) was calculated according to the concentrations with similar number of copies (beef: 342 copies/μL, mutton: 338 copies/μL, pork: 376 copies/μL, chicken: 356 copies/μL, and duck: 326 copies/μL) of diluted DNA templates and mixed in the same proportion. For single templates, multiply the concentration by percentage. For mixed templates, average the concentrations and multiply by percentage.

Five pairs of primers and probes were added to the same PCR system to establish quintuple ddPCR. In order to study the accuracy of the quintuple ddPCR system, the known components of DNA were detected. The DNA template was used to simulate the mixture of meat products. The results are shown in Table 7. Firstly, the setting proportion of beef DNA in the mixed DNA templates assays were 95%, 50% and 5%, respectively, with measured values of 92.4%, 52.2% and 3.8% respectively. Meanwhile, the measured values of number of copies of mutton DNA in the mixed templates were 94.8%, 49.5% and 5.4%, respectively. Finally, the measured values of beef and mutton combination in the mixed templates were 96.3%, 51.4% and 5.1%, respectively.

## 4. Discussion

qPCR is widely used in meat species identification and other food-safety component detection [7,18,19]. However, the standard curve needs to be established and mutual interference between primers and probes is inevitable and dramatically affects the application of the assay.

Although other assays are available for rapid qualitative detection, such as LAMP (loop-mediated isothermal amplification) [11,20] and RPA (recombinase polymerase amplification) [21], these detection methods cannot accurately quantify the adulterants or distinguish between intentional incorporation and careless contamination [22,23]. Since ddPCR is suitable for establishing multiple detection systems because of the multiple liquid separation of the initial amplification and the mutual interference between primers is effectively avoided by droplet formation [24], it is considered the most efficient technique for accurate quantitative analysis by far.

Cai et al. [9] developed a duplex droplet digital PCR detection and quantification system to simultaneously identify and quantify the source of meat in samples containing mixture of beef (*Bos taurus*) and pork (*Sus scrofa*). The results of the experiment validated that the system had positive practicability. Beef and mutton were also mixed with duck and chicken [1]. Two kinds of detection systems need to be established to perform the component detection of beef and mutton products. In order to simplify the operation process and improve the detection efficiency, a multiplex ddPCR system was established to detect five meat ingredients (beef, mutton, pork, duck and chicken). It can not only detect whether pork, chicken and duck are mixed in beef or mutton products at one time but also provide reference to judge whether it is contamination or intentional incorporation by quantitative analysis.

Although high amplification efficiency of primers is not required using ddPCR, in the actual detection, we found that the low amplification efficiency of primers would have a negative impact on the results. Accordingly, we used qPCR to detect the amplification efficiency of primers in this study. Although ddPCR can avoid the interference between primers and probes in theory, we studied the interference between primers and probes by adding primers step by step. The results showed that there was a certain degree of interaction between primers, but the results were very close to the theoretical value. At the same time, the effect of template concentration on the results was also investigated. The results indicated that the template concentration was between 10 ng/µL and 20 ng/µL, and the value of detection was relatively accurate.

Compared with experimental data from ddPCR studies [18], no significant variation of the species-specific target gene copy number, as measured by the multiplex ddPCR assays, was observed. Based on the specificity, repeatability, consistency, limit of detection, and limit of quantification of the assay, the multiplex ddPCR assay has comparable detection performance to ddPCR. The multiplex ddPCR assay has the advantages of reducing cost and time required for quantification of meat composition in complex samples. Similar experimental conclusions have been reported in other literature [12].

## 5. Conclusions

Here, we designed five pairs of primers for ddPCR of specific genomic region from five selected species where specificity and amplification efficiency was determined. The multiplex ddPCR method demonstrated good performance in identifying the ingredients in mixed samples based on DNA content, indicating that the technique has the potential to facilitate screening for food adulteration and mislabeling in other food products.

## Figures and Tables

**Table 1:** Primer and probe sequences.

Name	Primers and Probes	Base Sequence (5′ to 3′)
*Bos taurus*	F	ATACTCCATCCAGAACACCCAG
	R	ATGCGAAGCAGCTCCAAGT
	P	HEX-CTTCTCTGAAACCATC-MGB
*Ovis aries*	F	CAGCCCTCGCCATAGTTCAC
	R	TTGTCTGGGTCTCCGAGTAAGTC
	P	HEX-TCTTCCTCCACGAAACAGGATCCAACA-MGB
*Anas platyrhynchos*	F	GATTCTACTTCACCGCCCTAC
	R	CTACGAAGTGTCAGTATCAGGC
	P	FAM-ATCCACCTTCCTAACCGTCTGCC-MGB
*Sus scrofa*	F	GGAGTGTGTATCCCGTAGGTG
	R	CTGGGGACATGCAGAGAGTG
	P	FAM-TCTGACGTGACTCCCCGACCTGG-MGB
*Gallus gallus*	F	AAGTGCTGGCTGTGAGTTGG
	R	CGCTCCGCTACCTAATTCCT
	P	FAM-CTGTACCTTAAGCCTGCTCAGACTCTGG-MGB

**Table 2:** Reaction system of ddPCR (20 µL).

Reaction Components	Volume (µL)			
	Double	Triple	Quadruple	Quintuple
ddPCR Supermix for Probes (No dUTP)	10	10	10	10
*Bos taurus*-F/R/P (10 µM each)	1 + 1 + 0.5	—	0.8 + 0.8 + 0.4/—	0.6 + 0.6 + 0.3
*Ovis aries*-F/R/P (10 µM each)	1 + 1 + 0.5	—	—/0.8 + 0.8 + 0.4	0.6 + 0.6 + 0.3
*Sus scrofa*-F/R/P (10 µM each)	—	0.8 + 0.8 + 0.4	0.8 + 0.8 + 0.4	0.6 + 0.6 + 0.3
*Gallus gallus*-F/R/P (10 µM each)	—	0.8 + 0.8 + 0.4	0.8 + 0.8 + 0.4	0.6 + 0.6 + 0.3
*Anas platyrhynchos*-F/R/P (10 µM each)	—	0.8 + 0.8 + 0.4	0.8 + 0.8 + 0.4	0.6 + 0.6 + 0.3
Template	1	1	1	1
ddH_2_O	4	3	1	1.5

**Table 3:** Determination of DNA concentration.

ID	Sample Type	260Abs (10 mm)	280Abs (10 mm)	260/280	Conc. (ng/μL)
*Bos taurus*	dsDNA	6.394	3.42	1.87	319.68
*Ovis aries*	dsDNA	5.151	2.764	1.86	257.53
*Sus scrofa*	dsDNA	5.892	3.178	1.85	294.6
*Gallus gallus*	dsDNA	10.364	5.674	1.83	318.19
*Anas platyrhynchos*	dsDNA	6.055	3.238	1.87	299.77

**Table 4:** Standard curves of five species.

Species	Regression Equation of Standard Curve	R^2^	Standard Error
*Bos taurus*	Ct = −3.702x + 30.76	0.9994	0.0406
*Ovis aries*	Ct = −3.321x + 28.15	0.9995	0.0235
*Sus scrofa*	Ct = −3.404x + 29.74	0.9994	0.0253
*Gallus gallus*	Ct = −3.420x + 30.23	0.9994	0.0280
*Anas platyrhynchos*	Ct = −3.401x + 29.12	0.9990	0.0474

**Table 5:** Measured values of ddPCR for beef and mutton.

DNA Template	Primers and Probe	Theory Value (Copies/μL)	Detection Value (Copies/μL)	Deviation
beef:mutton (1:1)	beef	171	164	−0.04
	mutton	169	166	−0.02
	beef and mutton	340	319	−0.06

**Table 6:** Measured values of ddPCR for pork, chicken and duck.

DNA Template	Primers and Probe	Theory Value (Copies/μL)	Detection Value (Copies/μL)	Deviation
pork:chicken:duck (1:1:1)	pork	125	132	0.06
	chicken	119	114	−0.04
	duck	109	97	−0.11
	pork, chicken and duck	353	323	−0.08
pork:chicken:duck (1:2:3)	pork	63	65	0.03
	chicken	119	106	−0.11
	duck	163	162	−0.01
	pork, chicken and duck	345	330	−0.04
pork:chicken:duck (1:3:6)	pork	38	35	−0.08
	chicken	107	104	−0.03
	duck	196	201	0.03
	pork, chicken and duck	341	316	−0.07

**Table 7:** Measured values of ddPCR for five species.

Template			Theory Value (Copies/μL) (HEX)	Detection Value (Copies/μL) (HEX)	Theory Value (Copies/μL) (FAM)	Detection Value (Copies/μL) (FAM)	Proportion			Deviation		
Beef	Mutton	Pork Chicken Duck					Beef	Mutton	Pork Chicken Duck	Beef	Mutton	Pork Chicken Duck
95%	0	5%	325	271	18	22	92.4%	0	8.6%	−0.03	0	0.72
50%	0	50%	171	149	176	136	52.2%	0	47.8%	0.04	0	−0.04
5%	0	95%	17	12	335	298	3.8%	0	96.2%	−0.24	0	0.01
0	95%	5%	321	254	18	15	0	94.8%	5.2%	0	0.00	0.04
0	50%	50%	169	152	176	138	0	52.4%	47.6%	0	0.05	−0.05
0	5%	95%	17	15	335	263	0	5.4%	94.6%	0	0.08	0.00
95%		5%	323	261	18	10	96.3%		3.7%	0.01		−0.26
50%		50%	170	146	176	138	51.4%		48.6%	0.03		−0.03
5%		95%	17	13	335	244	5.1%		94.9%	0.02		0.00

## Data Availability

Data is contained within the article.
